# Icaritin Derivative IC2 Induces Cytoprotective Autophagy of Breast Cancer Cells via SCD1 Inhibition

**DOI:** 10.3390/molecules28031109

**Published:** 2023-01-22

**Authors:** Yi-Xuan Wang, Yi-Yuan Jin, Jie Wang, Zi-Cheng Zhao, Ke-Wen Xue, He Xiong, Hui-Lian Che, Yun-Jun Ge, Guo-Sheng Wu

**Affiliations:** 1 Department of Basic Medical Science, Wuxi School of Medicine, Jiangnan University, Wuxi 214000, China; 2 Taizhou Center for Disease Control and Prevention, Taizhou 318000, China

**Keywords:** icaritin, SCD1, autophagy, breast cancer

## Abstract

Breast cancer is one of the most prevalent malignancies and the leading cause of cancer-associated mortality in China. Icaritin (ICT), a prenyl flavonoid derived from the *Epimedium Genus*, has been proven to inhibit the proliferation and stemness of breast cancer cells. Our previous study demonstrated that IC2, a derivative of ICT, could induce breast cancer cell apoptosis by Stearoyl-CoA desaturase 1 (SCD1) inhibition. The present study further investigated the mechanism of the inhibitory effects of IC2 on breast cancer cells in vitro and in vivo. Our results proved that IC2 could stimulate autophagy in breast cancer cells with the activation of adenosine monophosphate (AMP)-activated protein kinase (AMPK) signaling and mitogen-activated protein kinase (MAPK) signaling. Combination treatment of the AMPK inhibitor decreased IC2-induced autophagy while it markedly enhanced IC2-induced apoptosis. In common with IC2-induced apoptosis, SCD1 overexpression or the addition of exogenous oleic acid (OA) could also alleviate IC2-induced autophagy. In vivo assays additionally demonstrated that IC2 treatment markedly inhibited tumor growth in a mouse breast cancer xenograft model. Overall, our study was the first to demonstrate that IC2 induced cytoprotective autophagy by SCD1 inhibition in breast cancer cells and that the autophagy inhibitor markedly enhanced the anticancer activity of IC2. Therefore, IC2 was a potential candidate compound in combination therapy for breast cancer.

## 1. Introduction

Cancer cells have a high demand for metabolites such as glucose and lipids; the latter especially play an important role as a source of energy and contribute to membrane building, as well as serving as secondary messengers for multiple molecular pathways [1,2]. SCD1, a rate-limiting enzyme which catalyzes the conversion of saturated fatty acids (SFA) to monounsaturated fatty acids (MUFA) in the fatty acid synthesis pathway [3], is involved in the occurrence and development of various tumors such as lung cancer, breast cancer, prostate cancer, renal cell carcinoma and hepatocellular carcinoma [4,5,6,7,8,9]. Furthermore, over-expressed SCD1 in cancer cells enriches membrane phospholipids with MUFA, producing a more fluid lipid membrane, and induces cancer cell proliferation, migration and invasion [4]. Our previous study also revealed that SCD1 was overexpressed and correlated with poor prognosis in breast cancer patients, and could be a novel therapeutic target for breast cancer [10].

Autophagy is an evolutionarily highly conserved intracellular catabolic pathway that engulfs and degrades part of the intracellular components through lysosomes, which is divided into several sequential steps, including nucleation, elongation, closure, fusion and degradation [11]. Abnormal autophagy is closely related to a variety of pathological processes such as tumors, neurodegenerative diseases, metabolic diseases, pathogen infections, etc. [12,13]. Interestingly, autophagy is assumed to actively participate in the regulation of infinite proliferation and apoptosis of cancer cells. It appears that autophagy plays a paradoxical role during cancer progress as it both suppresses tumorigenesis and supports the survival of established tumors [14,15]. There are also some connections between SCD1 and autophagy [11]. It was reported that knocking out the SCD1 homolog gene Desat1 in Drosophila blocked the occurrence of autophagy [16], and the suppression of SCD1 expression contributed to the induction of autophagy in both HepG2 and SMMC-7721 cells [9]. Moreover, SCD1 may be an autophagy-related protein in non-small cell lung cancer, and the combination therapy of autophagy inhibition and SCD1 inhibition could achieve a synergistic antitumor effect in vitro and in vivo [17].

*Epimedium Genus* is derived from the Berberidaceae family and is used as a traditional Chinese medicine to strengthen the muscles and bones, tonify kidney yang and dispel wind and dampness [18]. ICT can be extracted from the dry stems and leaves of *Epimedium Genus* directly or produced by hydrolyzing the other major flavonoids in *Epimedium Genus* [19]. Several methods including column chromatography, chemical synthesis, and acid and enzymatic hydrolysis have been developed for the production of ICT. Among these approaches, enzymatic hydrolysis is deemed to have great potential owing to its strict selectivity, mild reaction conditions, high efficiency and environmental friendliness [20]. ICT has been widely studied and proven to have rich medicinal value, including immune system regulation, antiinflammation, antitumor activities, neuroprotection, cardiovascular protection, bone metabolism, lipid metabolism regulation, etc. [21,22,23,24,25,26]. In breast cancer cells, ICT exerted antitumor effect by inducing cell cycle arrest at the G2/M phase and sustained phosphorylation of ERK [25]. Furthermore, ICT displayed anticancer activities by inhibiting IL-6/JAK/STAT3 pathways in advanced hepatocellular carcinoma cells and enhancing the effector T cell function [27]. Recently, ICT was approved by the National Medical Products Administration of China for the treatment of advanced hepatocellular carcinoma [19]. However, whether ICT-based derivatives could also have a better performance in cancer treatment is unclear.

In the previous study, we used computer-aided drug design technology to modify ICT and screened out the compound IC2 with a potential SCD1 inhibitory effect. Compared to ICT and other ICT derivates, IC2 showed the highest binding free energies (−114.9 kcal/mol) with SCD1, and the most significant inhibition of breast cancer cell proliferation. The subsequent experiments also confirmed that IC2 truly inhibited SCD1 activity in a dose-dependent manner and induced apoptosis in breast cancer cells [10]. Therefore, in this study, we further explored the mechanism of IC2-induced anti-breast cancer effect in vitro and in vivo, and we demonstrated that IC2 mediated cytoprotective autophagy in breast cancer cells through SCD1 inhibition, which provided a promising strategy for clinicians to explore the treatment of breast cancer.

## 2. Results

### 2.1. IC2 Induces Cell Autophagy in Breast Cancer Cells

**Figure 1:**
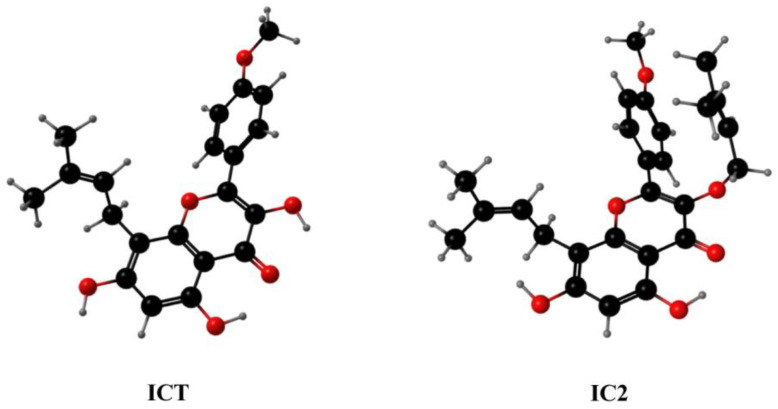
The three-dimensional chemical structures of ICT and IC2. Carbon is depicted with black balls. Oxygen is depicted with red balls. Hydrogen is depicted with gray balls.

**Figure 2:**
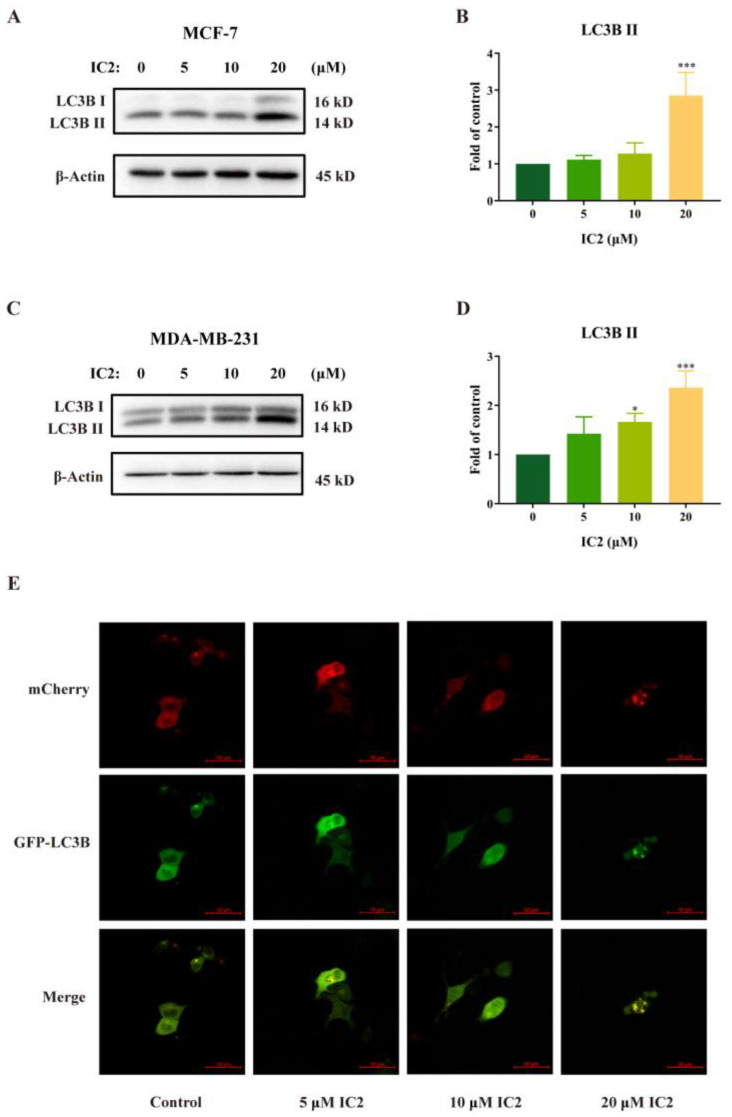
IC2 increases LC3B II expression and GFP-LC3 puncta formation in breast cancer cells. After IC2 treatment for 24 h, the expression of LC3B was determined in (**A**) MCF-7 cells and (**C**) MDA-MB-231 cells by Western blot. Quantification of the protein expression level of LC3B Ⅱ in (**B**) MCF-7 cells and (**D**) MDA-MB-231 cells. All blots are representative of at least three repeats. * *p* < 0.05, ** *p* < 0.01 and *** *p* < 0.001 compared with the control group. (**E**) Representative images of MCF-7 cells infected with Ad-mCherry-GFP-LC3B adenovirus after IC2 treatments at different doses. Images were at 200× magnification.

The ICT derivative IC2 (Figure 1) inhibited SCD1 activity in a dose-dependent manner and induced apoptosis by the mitochondrial pathway in MCF-7 cells, which has been demonstrated in previous studies [10]. To understand whether autophagy played an important role in IC2-induced cell death, we examined the related indicators of autophagy. Western blot results showed that the expression of LC3B II was gradually raised with the increase in IC2 concentration in MCF-7 cells (Figure 2A,B) and MDA-MB-231 cells (Figure 2C,D). In addition, after transfecting MCF-7 cells with Ad-mCherry-GFP-LC3B recombinant adenovirus, it can be observed under a fluorescence microscope that mCherry-GFP-LC3B aggregates on the autophagosome membrane after 20 μM IC2 treatment, which was shown as yellow puncta (Figure 2E). This further proved that IC2 could stimulate autophagy in breast cancer cells.

### 2.2. IC2 Induces Autophagy by AMPK/mTOR and MAPK Signaling Pathways in MCF-7 Cells

**Figure 3:**
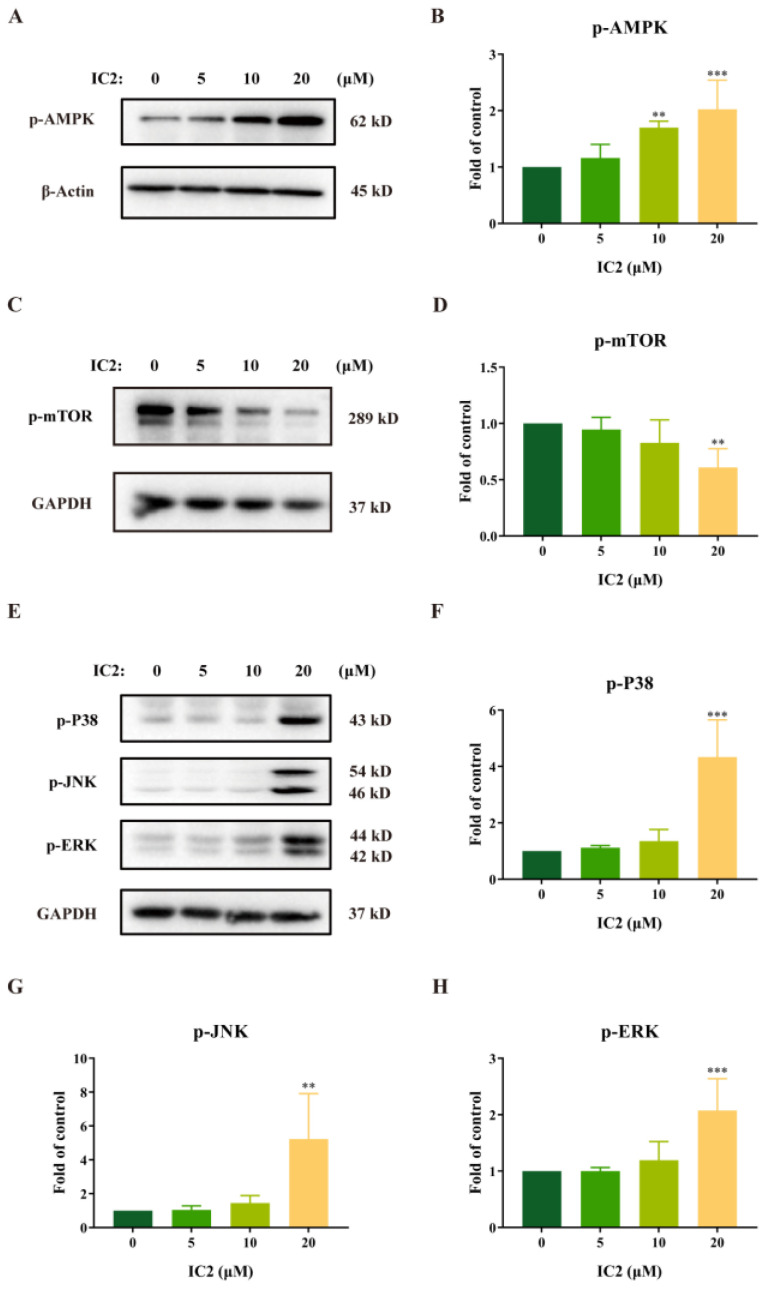
IC2-induced autophagy is correlated with the AMPK and MAPK signaling pathways. After IC2 treatment for 24 h, the expression of (**A**) p-AMPK (Thr172), (**C**) p-mTOR (Ser2448), (**E**) p-P38 (Thr180/Tyr182), p-JNK (Thr183/Tyr185) and p-ERK (Thr202/Tyr204) was determined in MCF-7 cells by Western blot. Quantification of the protein expression levels of (**B**) p-AMPK (Thr172), (**D**) p-mTOR (Ser2448), (**F**) p-P38 (Thr180/Tyr182), (**G**) p-JNK (Thr183/Tyr185) and (**H**) p-ERK (Thr202/Tyr204) in MCF-7 cells. All blots are representative of at least three repeats. * *p* < 0.05, ** *p* < 0.01 and *** *p* < 0.001 compared with the control group.

External stimuli such as oxidative stress, starvation, and cold can induce autophagy activity. The AMPK/mTOR signaling pathway is closely related to the enhancement of autophagy and is always activated in cancers [28,29]. To further explore the molecular mechanism of IC2 on induction of autophagy in MCF-7 cells, we assessed its effects on the autophagy-related signaling pathways. From our experimental results, it can be found that IC2 dose-dependently promoted the phosphorylation of AMPK (Figure 3A,B), and additionally inhibited the phosphorylation of mTOR in a dose-dependent manner (Figure 3C,D). Therefore, it can be inferred that the AMPK/mTOR pathway in MCF-7 cells may contribute to IC2-induced autophagy.

A large number of studies have shown that the MAPK family plays an important role in the process of cell growth and death, and that ICT can also exert an antitumor effect in breast cancer cells by continuously activating ERK [25,30,31]. Our results demonstrated that the expression of p-P38, p-JNK and p-ERK was significantly up-regulated with the treatment of IC2 (Figure 3E–H), indicating that MAPK was likely to be involved in the induction of autophagy by IC2.

### 2.3. IC2 Induces Cytoprotective Autophagy and AMPK Is Involved in Regulating the Balance of Autophagy and Apoptosis

**Figure 4:**
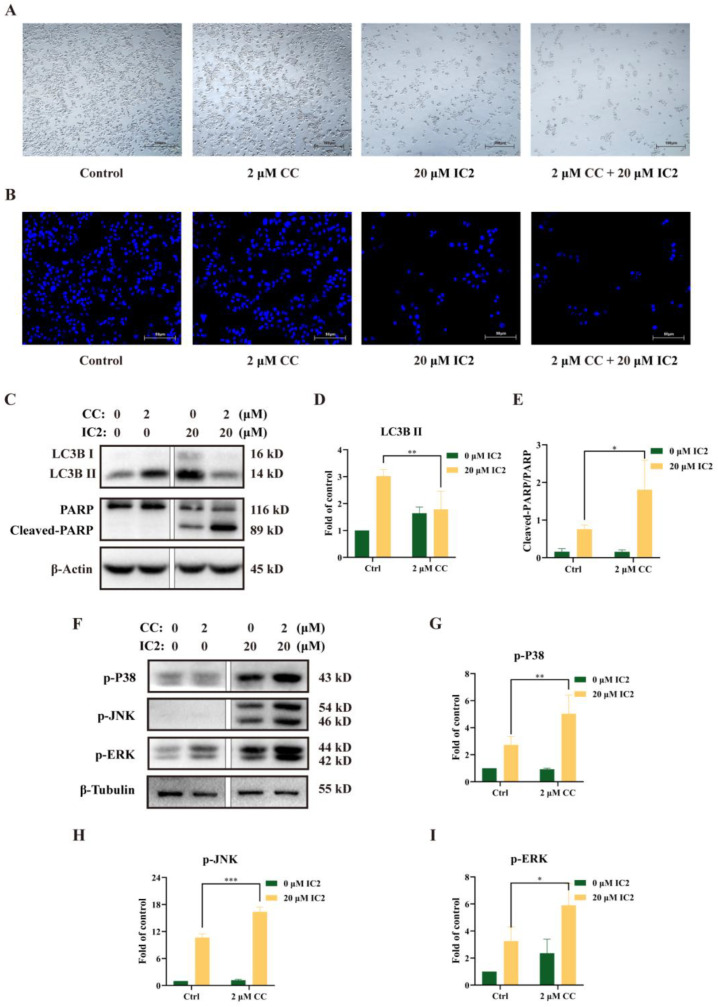
Inhibition of autophagy by AMPK inhibitor increases IC2-induced apoptosis and activation of MAPK signaling in MCF-7 cells. The cells were treated with IC2 for 24 h in the presence or absence of CC. (**A**) Representative images of MCF-7 cells were captured by brightfield microscopy at 50× magnification. (**B**) Representative images of DAPI staining were at 200× magnification in MCF-7 cells. The expression of (**C**) LC3B, PARP, (**F**) p-P38 (Thr180/Tyr182), p-JNK (Thr183/Tyr185) and p-ERK (Thr202/Tyr204) was determined in MCF-7 cells by Western blot. Quantification of the protein expression levels of (**D**) LC3B Ⅱ, (**E**) Cleaved-PARP/PARP, (**G**) p-P38 (Thr180/Tyr182), (**H**) p-JNK (Thr183/Tyr185) and (**I**) p-ERK (Thr202/Tyr204) in MCF-7 cells. All blots are representative of at least three repeats. * *p* < 0.05, ** *p* < 0.01 and *** *p* < 0.001 compared with the control group.

In order to clarify whether there is a certain connection between the occurrence of autophagy and apoptosis in MCF-7 cells administrated with IC2, we used the AMPK inhibitor compound C (CC) to conduct follow-up experiments. Observation under the microscope showed that the combination of CC and IC2 reduced the number of viable cells (Figure 4A). DAPI staining further confirmed that CC significantly promoted IC2-induced apoptosis (Figure 4B). Compared with the IC2-treated group, Western blot analysis also showed that the expression of LC3B II was significantly decreased after the addition of CC (Figure 4C,D), while the expression of Cleaved-PARP/PARP was increased (Figure 4C,E), indicating that the combined treatment of the autophagy inhibitor markedly enhanced the anticancer activity of IC2. It also proved that AMPK was an important upstream molecule in IC2-induced autophagy and that IC2 mediated cytoprotective autophagy in breast cancer cells.

To further clarify whether the MAPK family is involved in the regulation of autophagy and apoptosis, we detected the expression of phosphorylation levels of P38, JNK and ERK after CC and IC2 treatment and found that blocking the autophagy by AMPK inhibition could increase the phosphorylation of MAPK-related proteins (Figure 4F–I).

### 2.4. IC2-Induced Autophagy Depends on the Inhibition of SCD1

**Figure 5:**
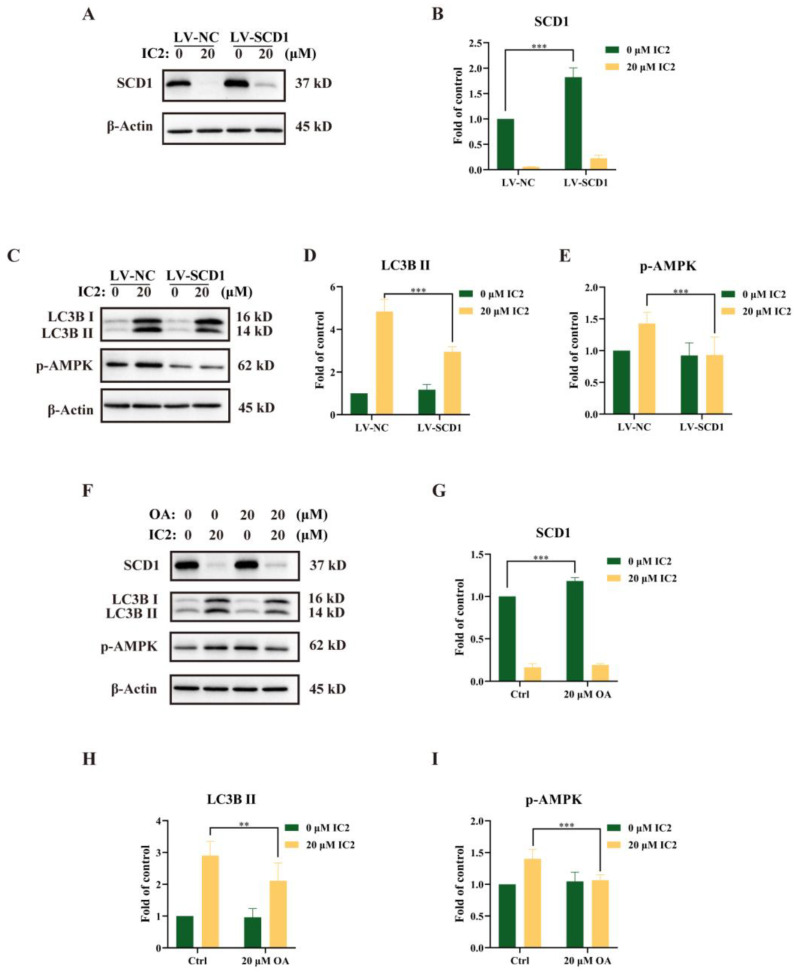
IC2-induced autophagy was alleviated by overexpression of SCD1 or exogenous addition of OA. (**A**) The expression of SCD1 was determined in corresponding normal MCF-7 cell line (LV-NC) and SCD1-overexpressing MCF-7 cell line (LV-SCD1) by Western blot. (**B**) Quantification of the protein expression level of SCD1 in LV-NC and LV-SCD1. (**C**) The expression of LC3B and p-AMPK (Thr172) was determined in LV-NC and LV-SCD1 by Western blot. Quantification of the protein expression levels of (**D**) LC3B Ⅱ and (**E**) p-AMPK (Thr172) in LV-NC and LV-SCD1. (**F**) The cells were treated with IC2 for 24 h in the presence or absence of OA in MCF-7 cells. The expression of SCD1, LC3B and p-AMPK (Thr172) was determined by Western blot. Quantification of the protein expression levels of (**G**) SCD1, (**H**) LC3B Ⅱ and (**I**) p-AMPK (Thr172) in MCF-7 cells. All blots are representative of at least three repeats. * *p* < 0.05, ** *p* < 0.01 and *** *p* < 0.001 compared with the control group.

Our previous study had revealed that IC2-induced antiproliferation effect and apoptosis were dependent on the inhibition of SCD1 expression and activity [10]. To further explore the relationship between SCD1 and IC2-induced autophagy in MCF-7 cells, we successfully constructed an SCD1-overexpressing MCF-7 cell line (LV-SCD1). IC2 treatment inhibited SCD1 expression in a corresponding normal MCF-7 cell line (LV-NC) and LV-SCD1 by Western blot (Figure 5A,B). After IC2 treatment, it can be observed that the expression of LC3B II and the phosphorylation of AMPK were alleviated after SCD1 overexpression (Figure 5C–E). In addition, we found that the expression of SCD1 was also increased after the addition of exogenous OA, a main product of SCD1 (Figure 5F,G). Consistent with the results above, the addition of OA decreased the expression of LC3B II and p-AMPK (Figure 5F,H,I). Together, these results indicated that SCD1 played an important role in the process of autophagy, which could alleviate IC2-induced autophagy.

### 2.5. IC2 Exerts Antitumor Activity in 4T1 Breast Cancer Xenograft Model

**Figure 6:**
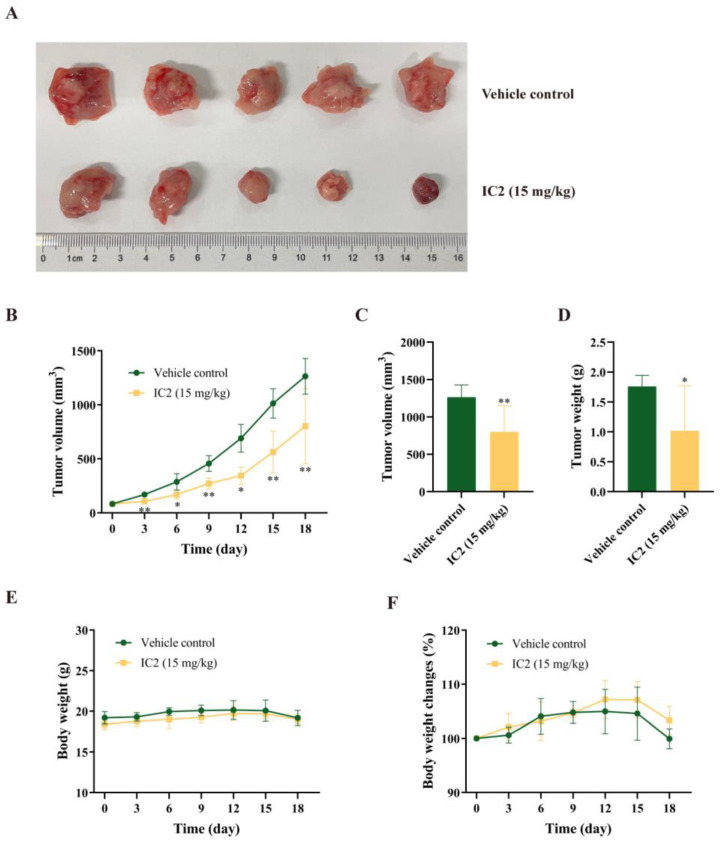
Antitumor activity of IC2 in 4T1 mouse xenograft model. (**A**) Representative photograph of removed tumor from the sacrificed mice at the end of the experiment. (**B**) Tumor volume (mm^3^) measured at indicated time points throughout treatment with IC2 (15 mg/kg). (**C**) Tumor volume (mm^3^) and (**D**) tumor weight at day 18 in groups treated with IC2 (15 mg/kg). (**E**,**F**) Body weight measured at indicated time points throughout treatment with IC2 (15 mg/kg). * *p* < 0.05, ** *p* < 0.01 and *** *p* < 0.001 compared with the control group.

In the previous study of our group, we found that IC2 induced apoptosis in breast cancer cells, but there is no in vivo demonstration. To determine whether IC2 would inhibit tumor growth, subcutaneous xenografts of the breast cancer cell line 4T1 were established in BALB/c mice. Compared to the control group, IC2 treatment resulted in a significant inhibition of tumor growth (Figure 6A,B). Correspondingly, the volume and weight of the tumor were significantly decreased compared to the control group under IC2 treatment. The average tumor volume in the IC2 (15 mg/kg)-treated group was 802.63 mm^3^, compared to 1264.85 mm^3^ in the control group, and the average tumor weight in the IC2-treated group was 1.02 g, compared to 1.76 g in the control group (Figure 6C,D).

There was no significant change in body weight between the two groups (Figure 6E,F). This indicated that IC2 had a positive orientation in the treatment of breast cancer and a certain potential for clinical application.

## 3. Discussion

At present, treatments for cancer include medication, radiotherapy and surgery, and most of the medication used to treat cancer consists of chemotherapy drugs, which are accompanied by drug resistance and toxic side effects [32]. In contrast, traditional Chinese medicine is multi-targeted, has low toxicity and is highly utilizable to selectively kill tumor cells and have a higher safety profile, making it an ideal choice for antitumor drug development. ICT is one of the active ingredients of the Chinese herb *Epimedium Genus*, and has been approved as a novel treatment for advanced hepatocellular carcinoma, demonstrating its efficacy, safety and broad therapeutic potential [19]. ICT truly exerts anti-hepatocellular carcinoma activity through various mechanisms including apoptosis, cell cycle regulation, anti-angiogenesis, anti-metastasis and immunomodulation [32,33]. In addition to being used in hepatocellular carcinoma treatment, ICT also has a good inhibitory effect on metabolism-sensitive cancers such as breast and prostate cancer [25,34]. Therefore, ICT should be chemically modified to expand the antitumor effect. Our previous study had identified that IC2 can markedly induce the apoptosis of breast cancer cells by SCD1 inhibition in vitro [10]. Herein, we proved that IC2 could exert an antitumor effect in the 4T1 breast cancer xenograft model, which further proves the clinical potential of IC2 as a candidate compound on SCD1-targeted therapy.

Human breast cancer is one of the leading causes of cancer-related death with few therapeutic treatment options in China. The role of autophagy in breast cancer is versatile and depends on the subtype of cancer cells and stage of the disease. Under a specific tumor microenvironment, autophagy is an important mechanism of metabolic adaptations to sustain the survival and proliferation of tumor cells. Therefore, a focus on more development and clinical tests for specific autophagy modulators is vital to provide a therapeutic benefit for breast cancer patients [35]. A previous study had demonstrated that ICT induced autophagy through upregulating the phosphorylation of AMPK and ULK1 in breast cancer cells [36]. Herein, similar to ICT, we found that IC2 increased autophagosome numbers and LC3B II expression in breast cancer cells by AMPK activation. Considering that IC2 has a better inhibitory effect on breast cancer cell proliferation than ICT [10], we then detected MAPK signaling after IC2 treatment in MCF7 cells and found that P38, JNK and ERK were significantly activated. Hence, based on our data, IC2 could be used as a potential autophagy inducer in breast cancer treatment.

Mounting evidence has indicated that autophagy acts as a “double-edged sword” during cancer progress, and that the relationship between autophagy and apoptosis is complex. Autophagy can induce apoptosis or adapt tumor cells to starvation and chemotherapy-induced damage, which contributes to cell survival [37]. Studies have proved that ICT-induced autophagy serves as either a facilitator or a blocker of apoptosis in different types of cancer cells. These conflicting data reflect the complex role of ICT in the regulation of autophagy in cancer cells [38]. Previously we have found that IC2 induced apoptosis in breast cancer cells via the mitochondrial pathway [10]. To clarify the interplay between IC2-induced autophagy and apoptosis, a combination of AMPK inhibitor CC and IC2 treatment was conducted. We found that this combination truly inhibited IC2-induced autophagy while it enhanced the cell apoptosis, which suggests that IC2-induced autophagy is cytoprotective. Furthermore, MAPK signaled after the combination of CC and IC2 treatment that P38, JNK and ERK were up-regulated accordingly. Given that IC2 had been proven to be an SCD1 inhibitor, we concluded that the combinational treatment of autophagy inhibition and SCD1 inhibition could be a promising strategy for breast cancer treatment.

SCD1, a lipogenic enzyme involved in the synthesis of MUFA from SFA, plays essential roles in promoting cancer cell proliferation and metastasis. Increasing evidence has indicated that autophagy and lipid metabolism are tightly interconnected in cancer cells, but the role of SCD1 as an autophagy inducer or inhibitor is not clear [11]. Herein, our results indicated that SCD1 functions as an autophagy inhibitor after IC2 treatment. IC2 has been shown to inhibit SCD1 activity and reduce the production of protective MUFA, while the accumulation of SFA leads to lipotoxicity to exert antitumor effects [39]. From the perspective of autophagy as a protective mechanism, the excessive accumulation of SFA may trigger an AMPK-mediated compensatory resistance due to the inhibition of SCD1, which is able to prevent further fatty acid synthesis while activating autophagy. This leads to a reduction in lipotoxicity, and an increase in cell viability. Perhaps targeting SCD1 could overcome the resistance mechanism in combination with an autophagy inhibitor, thereby promoting cell death [40].

In conclusion, we demonstrated for the first time that IC2 induces a cytoprotective autophagy dependent on AMPK signaling in breast cancer cells, and that SCD1 is critical for IC2-induced autophagy.

## 4. Materials and Methods

### 4.1. Experimental Reagents

ICT (Cat: E-0846) was purchased from Nanjing Spring & Autumn Biotech Co., Ltd. (Nanjing, China). Oleic acid (OA, Cat: A63262) was purchased from Sinopharm (Shanghai, China). Fetal bovine serum (FBS; Cat: 04-001-1ACS) was purchased from Biological Industries (Kibbutz Beit-Haemek, Israel). Ad-mCherry-GFP-LC3B (Cat: C3011), Penicillin-Streptomycin Solution (Cat: C0222) and Cell lysis buffer for Western and IP (Cat: P0013) were obtained from Beyotime (Shanghai, China). Dulbecco’s modified Eagle’s medium (DMEM; Cat: SH30022) and DAPI solution (Cat: MA0128) were purchased from Meilunbio (Dalian, China). The Pierce^TM^ BCA Protein Assay Kit (Cat: 23227) was purchased from Thermo (Waltham, MA, USA). All antibodies against β-actin (Cat: 4970T), β-Tubulin (Cat: 2128T), GAPDH (Cat: 5174T), LC3B (Cat: 12741T), p-AMPK (Cat: 2535T), p-mTOR (Cat: 5536T,), p-P38 (Cat: 4511T), p-JNK (Cat: 4668T), p-ERK (Cat: 4370T), PARP (Cat: 9542P), SCD1 (Cat: 2438S) and horseradish peroxidase-conjugated secondary antibody (Cat: 7074T) were purchased from Cell Signaling Technology (Beverly, MA, USA). Enhanced chemiluminescence (ECL) system (Cat: P10300) was purchased from NCM Biotech (Suzhou, China). All reagents used in the experiments were of analytical grade or higher.

### 4.2. Cell Culture

MCF-7, MDA-MB-231, and 4T1 cells were obtained from the Cell Bank of Shanghai Institutes for Biological Sciences, Chinese Academy of Sciences. MCF-7, MDA-MB-231, and 4T1 cells were cultured in DMEM with 10% FBS, 100 U/mL penicillin and 0.1 mg/mL streptomycin. Cells were maintained at 37 °C in a humidified environment in an incubator with 5% CO_2_.

### 4.3. Preparation of IC2

ICT and 1-bromo-3-methyl-2-butene were dissolved in 150 mL of acetone. Subsequently, 300 mg of anhydrous potassium carbonate was added to the mixture. The reaction mixture was heated to 56 °C and stirred for 4 h. The reaction mixture was then concentrated under reduced pressure, adjusted to pH 4–5 with 1 N HCl and then extracted three times with 100 mL of dichloromethane. The organic extracts were dried over anhydrous sodium sulfate, filtered and concentrated under reduced pressure. The resulting residue was purified by silica gel column chromatography (petroleum ether:ethyl acetate = 10:1) to obtain IC2 [10].

### 4.4. Western Blot

Cell lysis buffer for Western blot and IP containing protease inhibitors was used to extract proteins from cells, and the total concentration of proteins was determined using the BCA Protein Assay Kit. Equal amounts of protein samples were collected for electrophoresis with SDS–PAGE and then transferred to PVDF membranes (Bio-Rad, Hercules, CA, USA). After blocking with 5% non-fat milk, the membranes were treated with primary antibodies (1:1000 dilution) and subsequently with secondary antibodies (1:3000 dilution). Finally, membrane visualization was performed on an ECL system, and the protein bands were detected using Chemi-DOC MP (Bio-Rad, Hercules, CA, USA). The density of the immunoreactive bands was quantified using ImageJ software (National Institute of Health, Bethesda, MA, USA).

### 4.5. Autophagic Flux Observation

To analyze the process of autophagy, adenovirus expressing mCherry-GFP-LC3B fusion protein was used in MCF-7 cells according to the instructions from the manufacturer. Cells were planted in 6-well plates and then cocultured with Ad-mCherry-GFP-LC3B at a MOI of 60 when cells reached 30% or 40% density. After 24 h, the adenovirus was removed and treated with IC2. A fluorescence microscope (Ti-U; Nikon, Tokyo, Japan) was used to observe the fluorescence change in different groups in order to study the autophagic flux.

### 4.6. DAPI Staining

MCF-7 cells were planted in 6-well plates overnight. After incubation with IC2 at different concentrations for 24 h, cells were fixed with 4% formaldehyde for 20 min and then washed with phosphate-buffered saline (PBS). Cells were then treated with 4′,6-Diamidino-2-phenylindole dihydrochloride (DAPI; 10 µL/mL) for 10 min at room temperature in the dark. After discarding the DAPI dye, we washed the cells with PBS to remove the redundant fluorescent dye and then observed them under a fluorescence microscope (Ti-U; Nikon, Tokyo, Japan).

### 4.7. Lentiviral Overexpression Model

We used a lentivirus to establish SCD1 overexpression models. The SCD1 overexpression lentiviral vector was purchased from Hanbio Biotech (Shanghai, China), and was transduced into MCF-7 cells according to the manufacturer’s instructions. In short, cells were cultured in a 24-well plate overnight. Before infection, the virus liquid was thawed on ice in advance. After 24 h of infection, the virus-containing culture medium was replaced with a fresh culture medium. The SCD1-overexpression vector carries the GFP reporter gene, flag-tagged SCD1 gene and the puromycin resistance gene. The negative control vector carries only the GFP reporter gene and the puromycin resistance gene. After 48 h of infection, the GFP expression efficiency of the cells was observed under a fluorescence microscope (Ti-U; Nikon, Tokyo, Japan) to evaluate the virus infection efficiency. Subsequently, the MCF-7 stable transgenic cell line was screened by puromycin (1 μg/mL), and finally determined by Western blot.

### 4.8. Establishment of the 4T1 Breast Cancer Model

Sub-confluent 4T1 cells were harvested, washed once in serum-free DMEM, and resuspended in serum-free DMEM at a concentration of 5 × 10^6^ cells/mL. A hundred μL of the cell suspension was then implanted into the abdominal subcutaneous tissue of female BALB/c mice. Once the tumor volume reached approximately 100 mm^3^ (5–10 days after implantation), the mice were randomized to the control group (*n* = 5; intratumoral injection with vehicle) and the IC2 group (*n* = 5; intratumoral injection with 15 mg/kg IC2) for drug intervention once per three days. Vehicle control consisted of 10% DMSO, 40% PEG300, 5% Tween 80 and 45% saline. All mice were sacrificed and dissected 12 h after the last administration. Tumors were excised and weighed [41]. The tumor volume was measured every three days using the following formula: $V=0.5\times d_{1}^{2}\times d_{2}$, where *d*_1_ is the shortest diameter and *d*_2_ is the longest diameter [42].

### 4.9. Statistical Analysis

Results are presented as mean ± standard deviation (S.D.). One-way ANOVA and Student’s *t*-test were used to test for significance in the experiments. Data were analyzed with a GraphPad Prism 8.0 (GraphPad Software, San Diego, CA, USA). *p* < 0.05 was considered statistically significant.

## 5. Conclusions

**Figure 7:**
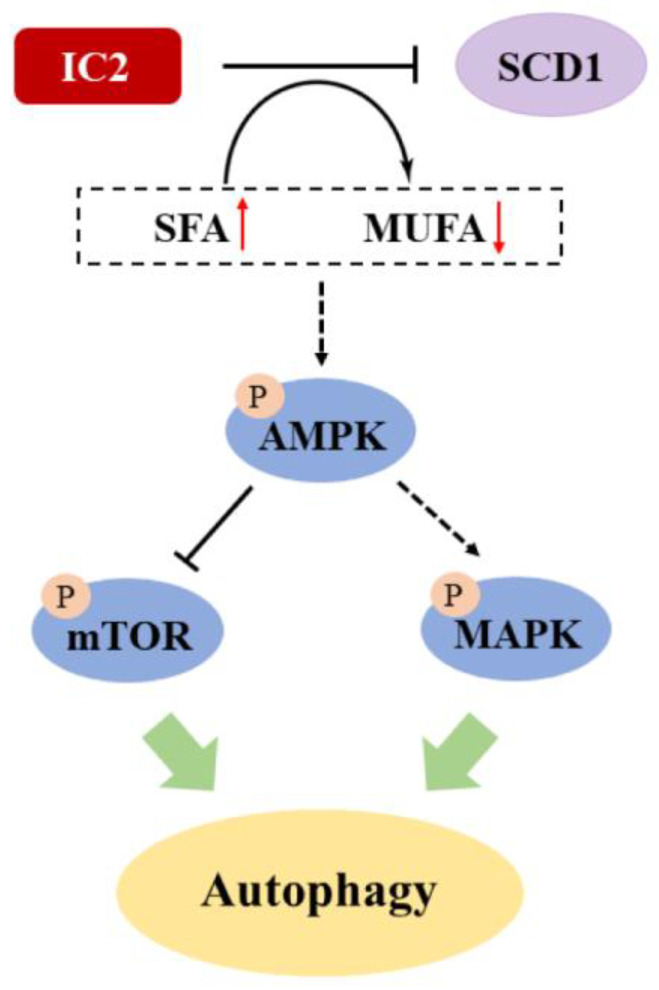
Proposed mechanism of IC2-induced autophagy in breast cancer cells. Solid lines indicate direct modification and dotted lines indicate tentative modification.

In this study, we found that IC2 could induce cytoprotective autophagy in breast cancer cells via the AMPK/mTOR- and MAPK-signaling pathways (Figure 7). SCD1 overexpression and exogenous addition of OA could alleviate IC2-induced autophagy. In addition, IC2 treatment-inhibited tumor growth in a mouse breast cancer xenograft model was determined by in vivo experiments. This study may provide insight into the clinical exploration of the combination of SCD1 inhibition and autophagy induction as well as the treatment of breast cancer. Further studies will focus on the specific mechanism of cancer lipid metabolism in IC2-induced autophagy and apoptosis, such as how SCD1 coordinate the interaction between autophagy and apoptosis by AMPK signaling. In addition, the combined use of the SCD1 inhibitor and the AMPK inhibitor against breast cancer is worth further investigation.

## Data Availability

The authors declare that the data supporting this study are available within the paper. All other data are available from the authors upon reasonable request.

## Sample Availability

Not available.
